# Viral vectors encoding endomorphins and serine histogranin attenuate neuropathic pain symptoms after spinal cord injury in rats

**DOI:** 10.1186/1744-8069-11-2

**Published:** 2015-01-07

**Authors:** Farinaz Nasirinezhad, Shyam Gajavelli, Blake Priddy, Stanislava Jergova, James Zadina, Jacqueline Sagen

**Affiliations:** Miami Project to Cure Paralysis, University of Miami Miller School Of Medicine, Miami, FL 33136 USA; Southeast Louisiana Veterans Health Care System (SLVHCS) and Senior Research Career Scientist Program (SRCS), Dept Medicine, Tulane University, New Orleans, LA 70112 USA; Physiology Research Center, Department of Physiology, Iran University of Medical Sciences, Tehran, Iran

**Keywords:** Central pain, Gene therapy, Lentiviral vectors, Endogenous opioids, NMDA antagonist, Antinociception, Spinal cord injury, Chronic pain

## Abstract

**Background:**

The treatment of spinal cord injury (SCI)-induced neuropathic pain presents a challenging healthcare problem. The lack of available robust pharmacological treatments underscores the need for novel therapeutic methods and approaches. Due to the complex character of neuropathic pain following SCI, therapies targeting multiple mechanisms may be a better choice for obtaining sufficient long-term pain relief. Previous studies in our lab showed analgesic effects using combinations of an NMDA antagonist peptide [Ser^1^]histogranin (SHG), and the mu-opioid peptides endomorphins (EMs), in several pain models. As an alternative to drug therapy, this study evaluated the analgesic potential of these peptides when delivered *via* gene therapy.

**Results:**

Lentiviruses encoding SHG and EM-1 and EM-2 were intraspinally injected, either singly or in combination, into rats with clip compression SCI 2 weeks following injury. Treated animals showed significant reduction in mechanical and thermal hypersensitivity, compared to control groups injected with GFP vector only. The antinociceptive effects of individually injected components were modest, but the combination of EMs and SHG produced robust and sustained antinociception. The onset of the analgesic effects was observed between 1–5 weeks post-injection and sustained without decrement for at least 7 weeks. No adverse effects on locomotor function were observed. The involvement of SHG and EMs in the observed antinociception was confirmed by pharmacologic inhibition using intrathecal injection of either the opioid antagonist naloxone or an anti-SHG antibody. Immunohistochemical analysis showed the presence of SHG and EMs in the spinal cord of treated animals, and immunodot-blot analysis of CSF confirmed the presence of these peptides in injected animals. In a separate group of rats, delayed injection of viral vectors was performed in order to mimic a more likely clinical scenario. Comparable and sustained antinociceptive effects were observed in these animals using the SHG-EMs combination vectors compared to the group with early intervention.

**Conclusions:**

Findings from this study support the potential for direct gene therapy to provide a robust and sustained alleviation of chronic neuropathic pain following SCI. The combination strategy utilizing potent mu-opioid peptides with a naturally-derived NMDA antagonist may produce additive or synergistic analgesic effects without the tolerance development for long-term management of persistent pain.

## Background

Long term treatment for neuropathic pain arising from spinal cord injury (SCI) remains an important health care problem [1–3]. The mechanisms underlying the development of such pain syndromes are complex and an area of active research [4–10]. SCI patients exhibit a relatively poor response to traditional analgesics such as opioids and ketamine [11, 12]. In addition the therapeutic doses are associated with adverse side-effects, especially during long-term use. As an alternate strategy to side-effect ridden monotherapy, additive or synergistic drug combinations that target multiple mechanisms is warranted. Such drug combinations could provide potent analgesia at lower doses while reducing side effects of high doses needed to achieve even modest antinociceptive effects in SCI patients. Numerous studies have shown enhanced antinociceptive effects of opioids when used in combination with NMDA antagonists, suggesting analgesic synergism [13–18]. Additionally reduced tolerance development to morphine and other μ-opioids has been obtained by combination with NMDA receptor antagonists [19–25]. Antinociceptive effects of intrathecally administered EM-1 can be significantly enhanced in a synergistic fashion by NMDA antagonists [26, 27]. Thus, the combined administration of opioids and NMDA antagonists is promising to manage difficult pain states. However, for long-term pain management, there are potential problems associated with the need for repeated bolus or continual delivery of exogenous pharmacologic agents which may be overcome by direct delivery of active drugs to a particular area of CNS via gene therapy. The goal of this study was to explore the potential for co-delivery of opioid-active peptides and an NMDA-active peptide via viral vectors to alleviate SCI pain on a sustained basis.

Endomorphins are amidated tetrapeptides originally isolated from bovine brain. They have high selectivity towards the μ-opioid receptor [28]. Compared to morphine, endomorphins show more potency in neuropathic pain conditions and may produce less undesired side effects [29]. These peptides show a potent, dose- and time-dependent antinociceptive effect after intraventricular and intrathecal injection in acute, neuropathic and inflammatory pain models [28, 30–32]. Histogranin is a 15-amino acid naturally occurring peptide which possesses post-synaptic NMDA receptor antagonist-like activity [33]. Findings in our laboratory suggest that SHG, a stable analogue of histogranin, attenuates NMDA-mediated nociception without producing neurotoxicity [34]. No attendant motor dysfunction was observed using a battery of tests, in contrast to findings with synthetic NMDA blockers [35, 36]. SHG also reduces the response of wide dynamic range neurons of the dorsal horn in response to C-fiber stimulation and enhances opioid analgesia [17, 37]. These findings suggest that SHG may be a useful adjunct for the long term management of conditions with persisting pain.

Long term transduction of non-dividing cells using lentiviral vectors is an attractive approach to achieve sustained release of therapeutic peptides in specific locations [38, 39]. With the advent of third-generation lentivirus vector technology, *in vivo* gene therapy approaches have become feasible [40]. This method uses the transfer plasmid pRRL that contains the enhancer and promoter from the U3 region of RSV joined to the R region of the HIV-1 LTR.

Because the first synapses between the primary nociceptor and the second order projection neuron are present in the dorsal horn, the spinal cord could be an attractive target to block nociceptive information and reduce the perception of pain. With this rationale, the goal of the present study was to determine whether the combination of lentiviral vectors for transfer of genes encoding endomorphins and SHG into the spinal cord would be a promising candidate therapy for attenuation of neuropathic pain following spinal cord injury [41].

## Results

### Expression of endomorphins and serine histogranin in neuroblastoma cell line

**Figure 1:**
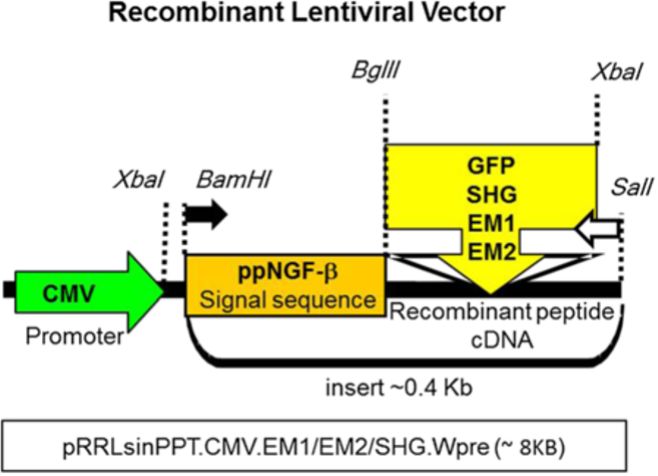
**Schematic of construct encoding recombinant peptides.** The schematic shows the features of the construct. The CMV promoter from lentiviral vector pRRL (pRRLsinPPT.CMV.EM1/EM2/SHG.Wpre) is followed by the signal sequence preproNGF-β (BamHI-BglII) and the cDNAs encoding for SHG or EM1 or EM2 (BglII-XbaI). The locations of forward and reverse gene specific primers used to assess gene expression are shown by filled and open arrows respectively.

**Figure 2:**
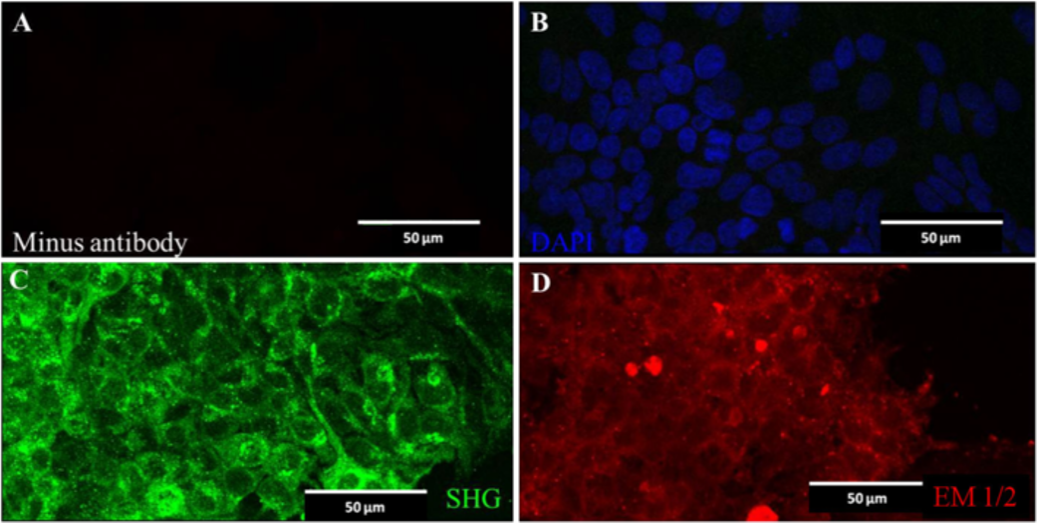
**Lentiviral transduction of neuroblastoma cell line.** Lentiviruses encoding SHG or Endomorphins were used to transduce a neuroblastoma cell line (SH-SY5Y). 5 days post-transduction cells were fixed and stained with anti-SHG or anti-endomorphins (1 and 2) antibodies. An image of negative control shows the absence of fluorescence **(A)**. The culture was counter stained DAPI (blue) to identify nuclei **(B)**. Green fluorescence in **(C)** and red fluorescence in **(D)** correspond to SHG and Endomorphin 1 or 2 respectively. Micron bar = 50 μm.

A schematic of the recombinant constructs is shown in Figure 1. To confirm the expression of the genes from the constructs, we used a human neuroblastoma cell line (SH-SY5Y). The transduction of the peptide encoding viral vectors was confirmed at five days by immunofluorescence. Using antibodies specific to endomorphins and serine histogranin, numerous positive cells were detected (Figure 2). The pattern of fluorescence of the peptide in the *in vitro* study was similar to that seen in the dorsal horn of spinal cord after injection of the vectors.

### Effects of viral injection on motor function

**Figure 3:**
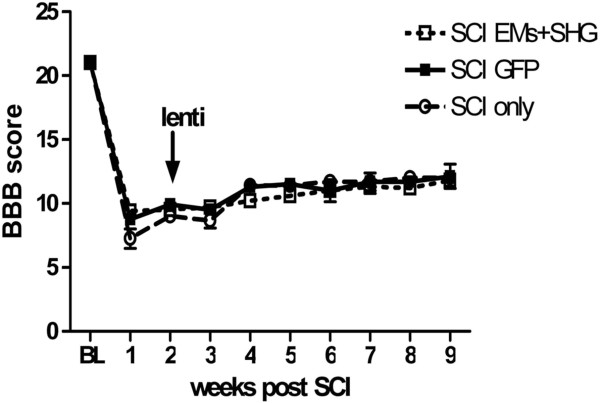
**Locomotor scores after lentiviral injection in SCI rats.** Effect of spinal cord injury (SCI) and injection of viral vector encoding GFP (n = 8) or a mixture of endomorphins and SHG (n = 10) injected two weeks after injury on BBB locomotor scores. Rats were tested prior to spinal compression (BL), one week after spinal compression (SCI), before viral injection and then weekly following injection for 7 weeks. Values are expressed as the mean ± S.E.M. No significant difference was observed between the two groups.

The effect of spinal cord injury and the intraspinal injection of vectors on motor function was evaluated by the BBB test (Figure 3). Before SCI the BBB locomotor score for rats was 21, indicating coordinated fore and hind paw movement, consistent toe clearance from the walking surface and trunk stability [42]. One week after SCI surgery, the BBB rating was about 9, which indicated plantar placement of the paw with weight support in stance only or stepping with the dorsal but not plantar surface of the hind paw. No further changes in locomotor function were observed two weeks following compression surgery. By 7 weeks after injection BBB scores increased to 11–12, which indicates frequent to consistent weight support with plantar steps and no or occasional fore-hindlimbs coordination. No significant differences were observed between the rats that received the mixture of viruses and the rats that were injected by control viruses for the 9 week observation period (P > 0.05). To verify if intraspinal injection itself has any effect on the motor behavior of the animals we evaluated the BBB scores in animals with SCI and without vector injection. The results showed no significant difference between this group of animals and animals receiving viral vectors. No other obvious side effects of the treatment were noted.

### Effects of viral injection on tactile and cold allodynia

**Figure 4:**
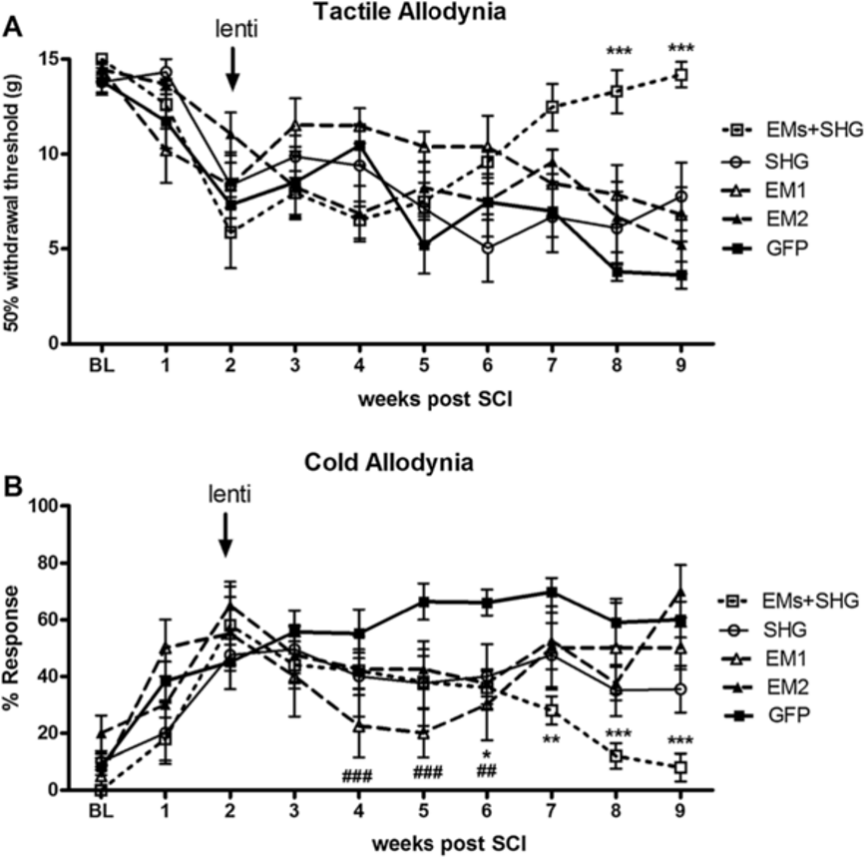
**Tactile and cold allodynia after lentiviral injection in SCI rats.** Tactile allodynia in the von Frey test **(A)** and cold allodynia in the acetone test **(B)** in SCI rats after lentiviral injection of GFP (n = 8), EM-1(n = 8), EM-2 (n = 8), SHG (n = 8) and EMs + SHG (n = 10) at 2 weeks after SCI. Values are expressed as the mean ± S.E.M. *p < 0.05; **p < 0.01; ***p < 0.001 for EMs + SHG vs GFP groups. ##p < 0.01; p < 0.001 for EM1 vs GFP groups.

Before SCI there were no significant differences among 5 groups in paw withdrawal threshold in the tactile allodynia test with von Frey filaments, or in the percentage of withdrawal responses in the acetone test for cold allodynia (Figure 4, P > 0.05). Figure 4A shows changes in von Frey responses over the 7 week time course following viral vector injections (overall F (df 4,9) = 7.941, P < 0.05). In this test, significant differences were observed by 6–7 weeks after injection (8–9 weeks post SCI) for the rats injected with the mixture of EMs and SHG compared to the control/GFP- injected animals (p < 0.001). No significant effects were observed among the other groups injected with single vectors or control GFP injected animals (Figure 4A, P > 0.05). Figure 4B shows changes in responses to cold (acetone test) over the 7 week time course following viral vector injections (overall F (df 4,9) = 3.875, P < 0.05). In the acetone test, significant differences were observed beginning at 2 weeks post injection in animals with EM-1 compared to GFP group. At the later time points, the mixture of EMs and SHG significantly reduced the pain related behavior compared to the other groups (P < 0.001 compared with control vector at 6–7 weeks after injection).

### Effects of viral injection on the mechanical and heat hyperalgesia

**Figure 5:**
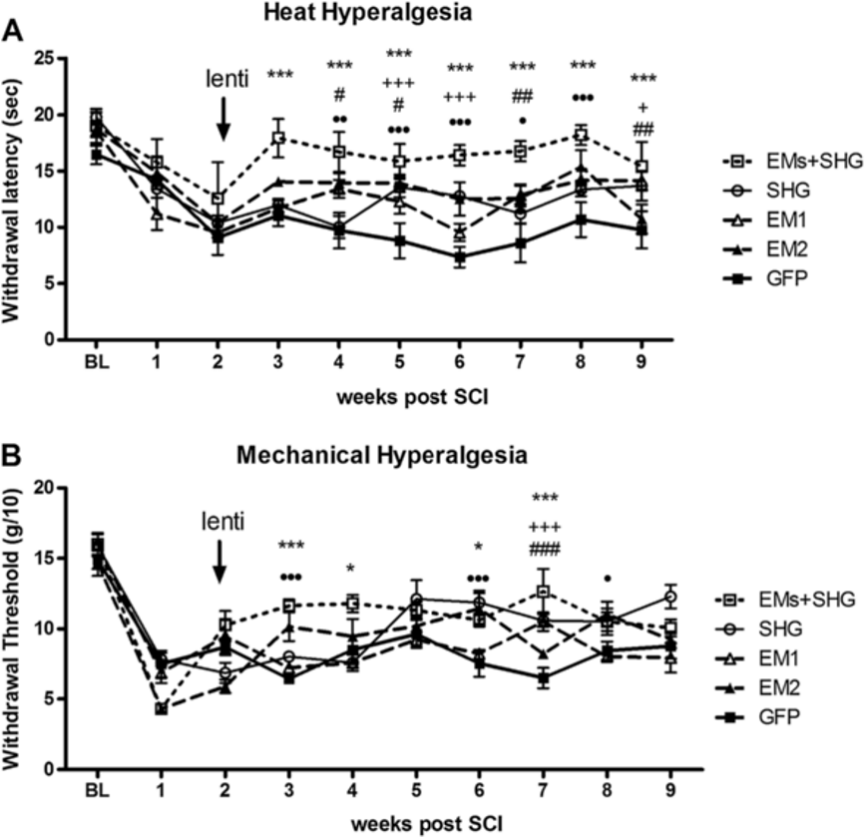
**Thermal and mechanical hyperalgesia after lentiviral injection in SCI rats.** Thermal hyperalgesia in the plantar test **(A)** and mechanical allodynia in the Randall-Selitto test **(B)** in SCI rats after lentiviral injection of GFP (n = 8), EM-1(n = 8), EM-2 (n = 8), SHG (n = 8) and EMs + SHG (n = 10) at two weeks after SCI. Values are expressed as the mean ± S.E.M. *p < 0.05; **p < 0.01; ***p < 0.001 for EMs + SHG vs GFP. +++p < 0.001; ++p < 0.01; +p < 0.05 for SHG vs GFP; ###p < 0.001; ##p < 0.01; #p < 0.05 for EM1 vs GFP. ∙∙∙p < 0.001; ∙∙p < 0.01; ∙p < 0.05 for EM2 vs GFP.

The Hargreaves plantar test was performed to determine whether viral injection affects thermal hyperalgesia. Before SCI surgery the paw withdrawal latency in all groups of animals was almost the same ranging from 16.48 ± 0.85 sec in control/GFP injected animal to 19.78 ± 0.45 sec in the group which received viral vectors encoding SHG (Figure 5A). No significant differences in baseline withdrawal latency were observed among animals in different groups (P > 0.05). Injection of the mixture of the vectors prolonged the paw withdrawal latencies beginning one week after injection and there was significant difference between this group with the control group injected with control/GFP (P < 0.001). The differences in the withdrawal latency between these two groups continued until the end of the experiment (P < 0.001). Attenuation of heat hypersensitivity was observed in all of the individual viral vector treated groups (SHG, EM-1, EM-2) compared to control GFP injection throughout the experiment (overall F(df 4,9) = 37.9; P < 0.05).

Mechanical nociceptive threshold, an index of mechano-hyperalgesia, was assessed by the pressure stimulation method as described by Randall and Selitto [43]. SCI resulted in the development of mechanical hyperalgesia as reflected by a significant decrease in the hind paw withdrawal threshold in the paw pressure test. A significant increase in mechanical threshold was observed between the mixed viral vector group and control animals injected with GFP vector during the 1–5 week post-injection points although this was modest and variable over time (Figure 5B). Significant differences between GFP and single-vector injected groups were observed as well with modest, but significant differences at some time points (overall F(df 4,9) =10.1).

### Effect of delayed injection of viral vectors on pain behavior

**Figure 6:**
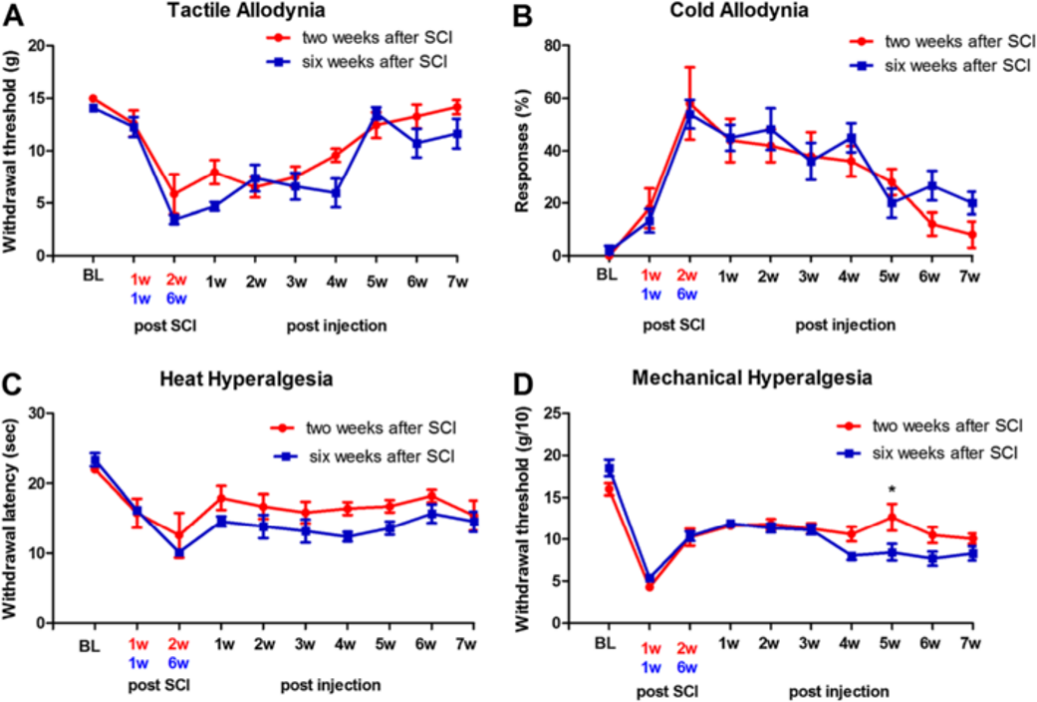
**Behavioral outcome of early and late injection of lentiviral constructs.** Tactile allodynia in the von Frey test **(A)**, cold allodynia in the acetone test **(B)**, thermal hyperalgesia in the plantar test **(C)** and mechanical hyperalgesia in the Randall-Selitto test **(D)** in SCI rats after viral injection encoding endomorphins + SHG injected at early (two weeks, n = 10) or late (6 weeks, n = 12) time points after SCI. Values are expressed as the ± S.E.M. There were no statistical differences observed between early and late SHG-EM injections comparing the data up to 7 weeks post injection, with the exception of week 5, when the threshold for mechanical allodynia was significantly higher in the group injected early post SCI. *p < 0.05.

To mimic clinical scenarios, the effect of time of delivery of therapeutic agents in chronically injured animals has to be considered. Therefore we evaluated the analgesic effect of intraspinal injection of our vectors at early (2 weeks) and delayed (6 weeks) time points post SCI. Results of this comparison are shown in Figure 6. The assessment of behavioral sensitivity after transplantation of the mixture of the vectors (SHG, EM-1, EM-2) showed nearly no differences in the ability to produce antinociception whether the vector administration occurred at 2 weeks or 6 weeks following injury (p > 0.05 for all behavioral tests). The only difference was detected at 5 weeks post injection for mechanical hyperalgesia, when animals injected at 2 weeks post SCI showed significantly higher sensitivity to mechanical pressure than animals injected at 6 weeks (p < 0.05). The reduced pain-related responses achieved in the animals receiving intraspinal injection of combined EMs and SHG by the end of the study (7 weeks following vector administration) was similar in both groups (p > 0.05).

### Effects of naloxone or SHG antibody on viral vector mediated attenuation of SCI allodynia

**Figure 7:**
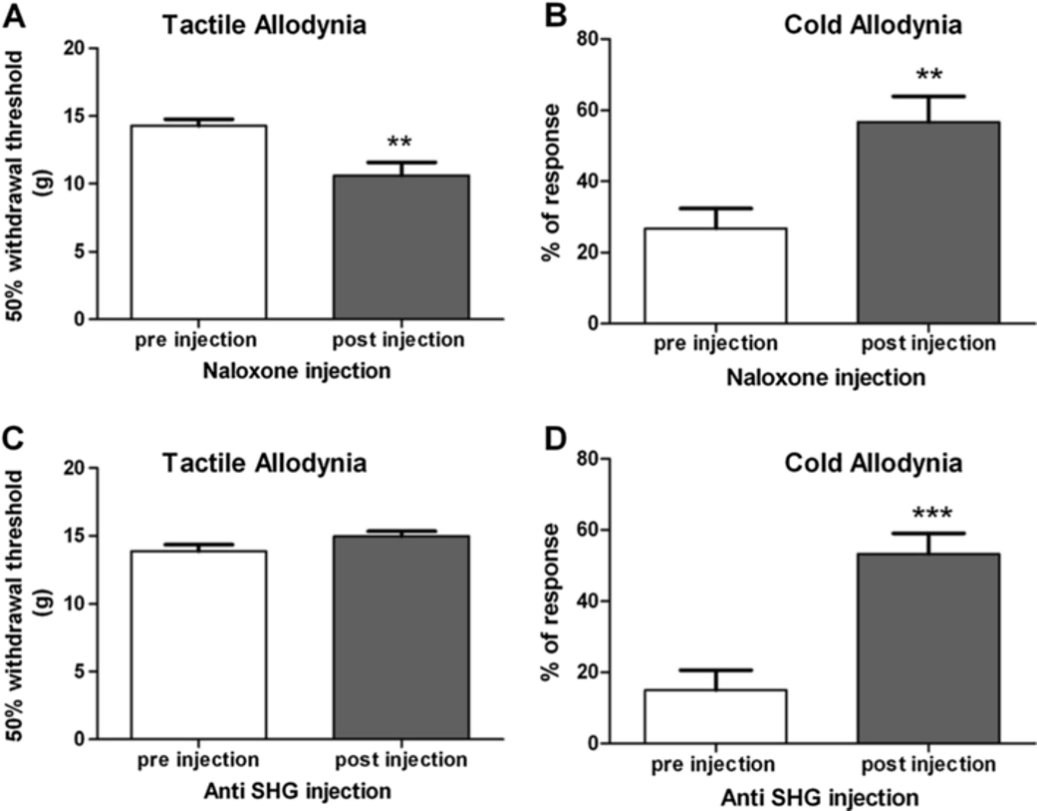
**Effect of Naloxone on tactile and cold allodynia in treated rats.** Mechanical allodynia **(A and C)** in the von Frey test and cold allodynia **(B and D)** in the acetone test before and after intrathecal administration of naloxone **(A and B)** and anti-SHG **(C and D)** in SCI animals treated 2 weeks after SCI with mixed EMs/SHG vectors. Values are expressed as the mean ± S.E.M. **P < 0.01, ***P < 0.001 compared to pre injection values. (n = 8, t-test).

The effects of intrathecal injection of the opioid receptor antagonist naloxone and an SHG antibody on the reduced cold and mechanical responses in animals with viral injection encoding endomorphins and SHG two weeks after SCI are shown in Figure 7. Naloxone reversed the antinociceptive effects of viral vectors, as indicated by a significant drop in withdrawal threshold (Von Frey test) and increased sensitivity to cold stimuli (acetone test) compared to pre-injection responses (P < 0.01, t-test). Injection of anti-SHG did not have any effect on the mechanical thresholds measured by von Frey filaments, but anti-SHG injection increased cold allodynia in treated animals (P < 0.001, t-test).

### Endomorphins and serine histogranin are expressed in dorsal horn of the spinal cord

**Figure 8:**
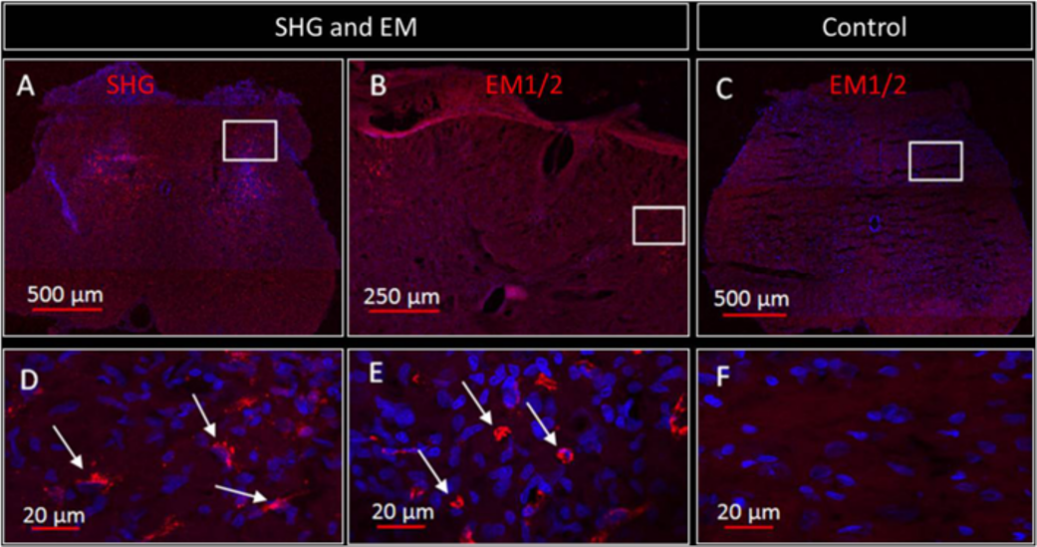
**Immunohistochemical detection of SHG and EMs in the spinal cord of treated rats.** Representative images of immunostained spinal cord sections from animal transduced with viral vector as indicated above the image **(A-B)** two weeks after SCI show expression (red fluorescence) of SHG **(A)** or EM 1/2 **(B)**. Spinal cord of a control untransduced animal immunostained with endomorphin antibodies is shown in **C**. Panels **D**, **E**, and **F** represent the higher magnification of rectangles of A, B and C, respectively. Arrows indicate some of the expressing cells.

**Figure 9:**
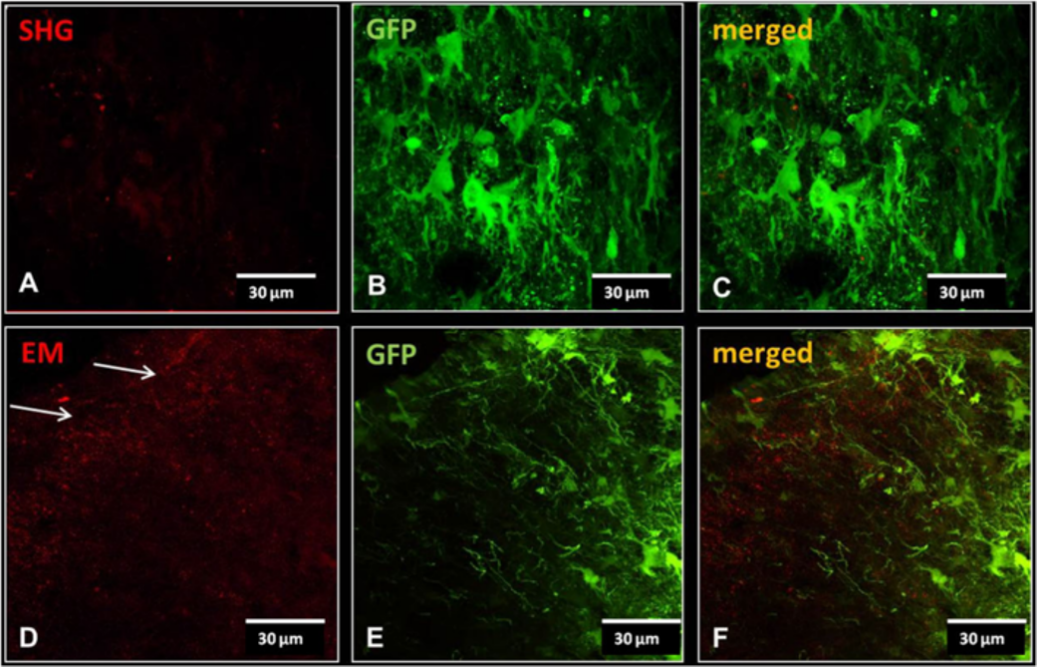
**Injection of control vector expressing GFP only.** No SHG signal was detected in spinal cords of animals receiving control GFP vector injection **(A)**. Staining with EM1/2 in these animals revealed punctate staining of the endogenous EM in the dorsal horn (arrows in **D**). **B** and **E** show the GFP fluorescence in cell bodies and processes in the same sections (**A** and **D**, respectively). **C** and **F** are merged images to reveal any co-localization.

Based on the behavioral results, we examined the expression of the endomorphins and serine histogranin in the dorsal horn of the spinal cord seven weeks after viral vector injection. Sections were labeled for endomorphins or SHG immunoreactivity. Immunoreactive positive cells for SHG (A) and endomorphins (B) were seen in the dorsal horn at the 7th week after viral injection (Figure 8). Peptide immunoreactivity appeared as light cytoplasmic immunostaining (red) with stronger intensity at the surface of the cell bodies of the transduced cells. No signal was found for SHG, and only low signal for EMs was observed in control animals without injection. In control animals receiving the control GFP viral vector injection, GFP fluorescence was apparent in cell bodies and processes in the injected spinal cords (Figure 9B and E), but there was no immunoreactivity for SHG (Figure 9A). Endogenous EM1/2 immunoreactivity was present in processes in the superficial dorsal horn (Figure 9D), but was not co-localized with the GFP (Figure 9F).

**Figure 10:**
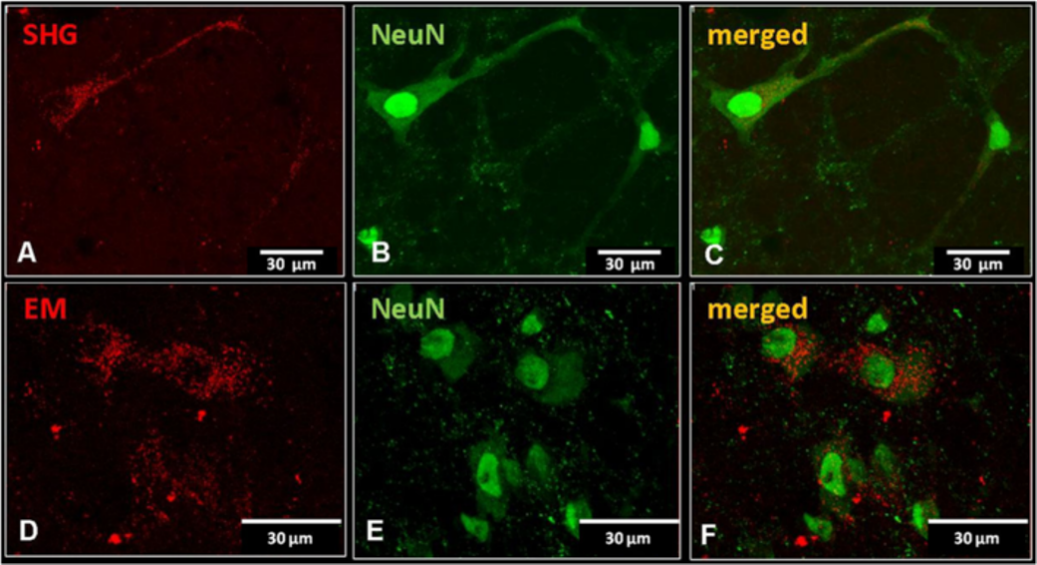
**Immunohistochemical detection of neuronal phenotype of transduced cells.** Co-localization of SHG **(A)** or EM2 **(D)** transduced cells expressing reporter gene mRFP (red) with neuronal marker (NeuN, green)) **(B,E)**. Overlapping images show the presence of punctate staining in the cytoplasm of neuronal cells **(C,F)**.

**Figure 11:**
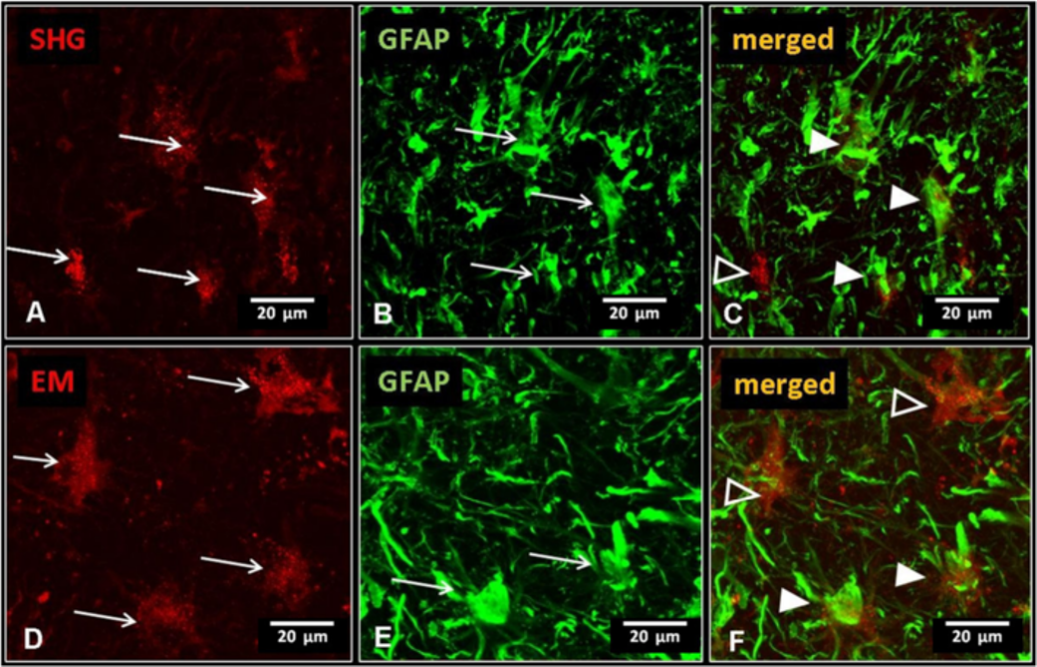
**Immunohistochemical detection of glial phenotype of transduced cells.** Cells positive for astrocytic marker GFAP **(B,E)** co-labeled with some of the SHG **(A-C)** or EM **(D-F)** transduced cells (full arrowheads in **C**,**F**). Open arrowheads mark non-GFAP positive cells transduced by SHG or EM respectively.

Additional staining was performed to detect possible phenotypes of the transfected cells at 2 weeks post injection of lentivectors with SHG or EM using mRFP (red) reporter. High magnification images show individual labelled cells following injection of Lenti/SHG (Figure 10A) or Lenti/EM-2 (Figure 10D), both with punctate distribution as found in the *in vitro* staining. A subpopulation of these cells co-localized with the neuronal marker NeuN (Figure 10B and E; co-localization in Figure 10 C and F).In the spinal cord of animals injected with either SHG or EM lentivectors (Figure 11A and D, respectively), co-localization was also observed in some astrocytes (GFAP; Figure 11B and E; merged with mRFP reporter in Figure 11C and F).

These data suggest that lentivectors used in this study transfect both neuronal and non-neuronal cell populations. However, based on our observations thus far, it appears that neuronal cells are transfected at higher levels than glial cells.

### Immunodot-blot analysis

**Figure 12:**
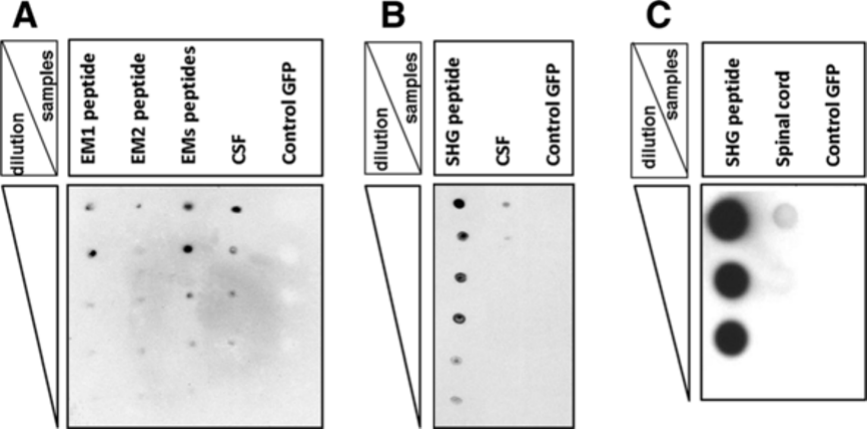
**Immunodot-blot analysis of CSF and spinal cord of treated rats.** Immunodot-blot assay of CSF from the GFP control SCI animals and SCI animals injected by a mixture of viral vectors encoding SHG and EMs. No reactivity was seen in the CSF of GFP control SCI animals. The amount of each peptide spotted is indicated in the column on the left side of each panel. **A**: reactivity of CSF with endomorphin 1 and 2 antibody, **B**: reactivity of CSF with SHG antibody. **C**: detection of SHG in the spinal cord homogenate from EMs + SHG treated animals.

The immunodot-blot analysis (Figure 12) indicated the presence of EMs (A) and SHG (B) in cerebrospinal fluid of the animals injected with the viral vectors and the presence of SHG in the spinal cord tissue of treated animals (C). The intensity of the immunoreactive spots showed a concentration dependency and was proportional to the amount of the peptides. These results confirm that the protein can be secreted following expression of the transgene by transduced cells in the spinal cord.

## Discussion

In this study, we have evaluated the analgesic properties of a combination of virally encoded opioid peptides EM-1, EM-2 with the NMDA antagonist SHG after their intraspinal injection. The expression was similar to that seen previously with these constructs in different cells [44]. The results showed that intraspinal delivery of the viral vectors mixture produced marked attenuation of neuropathic pain syndromes which lasted up to two months post injection. Antinociceptive effects of intraspinal injection of lentiviruses encoding EMs and SHG on mechanical and cold allodynia appeared to develop with a gradual onset by five weeks after injection. In contrast, heat and mechanical hyperalgesia attenuation were achieved by the first week following injection. Early transgene expression following intra-spinal lentivirus injection has been reported in other studies. The antinociceptive effects were sustained through the remainder of the study (7 weeks) and could be transiently reversed by intrathecal administration of anti-serine histogranin antibody and the opiate antagonist naloxone, suggesting the participation of opioid and NMDA receptors in the observed effects.

Gene therapy has several benefits compared to traditional pharmacological therapy. The use of viral vectors offers a potential therapeutic option for treatment of central nervous system disorders. Gene transfer approaches may be a useful method to achieve continuous local expression of short lived analgesic peptides in the spinal cord and are being considered for clinical application. Many of the previous studies have utilized herpes simplex viruses (HSVs) as a delivery vector in pain treatment. In the rodent CCI neuropathic pain model, an increase in paw withdrawal latencies to mechanical and thermal stimulus following application of recombinant defective herpes simplex viral vector encoding proenkephalin has been reported [45]. Also subcutaneous injection of HSV encoding preproenkephalin to the plantar surface of the paw increased the nociceptive threshold in phase II of the formalin test [46].

Recently, a Phase I clinical trial of HSV-mediated gene transfer of proenkephalin to the DRG was reported to produce significant pain relief in patients with cancer pain [47]. Endomorphin-2 has also been successfully delivered using HSV to reduce both inflammatory and peripheral neuropathic pain in rats [48, 49].

In addition to HSV vectors, pain modulator genes have been delivered directly to the spinal cord using adeno-associated viruses (AAVs) and lentiviruses [50–53].

The possibility of long-term expression of therapeutic peptides in specific cells is especially attractive as a treatment option for conditions such as SCI neuropathic pain. Neuropathic pain is initiated or caused by multiple etiological factors; therefore sustained simultaneous targeting of several signalling pathways is likely necessary to obtain effective analgesia. Lentiviral delivery is advantageous in that both dividing and non-dividing cells can be reached; it confers long-term stable expression of transgenes encoding naturally occurring peptides without detectable pathology and shows low immunogenicity [54, 55]. In other studies using lentiviruses, no increase in inflammatory markers like interleukin-mRNA was seen [56][50]. Considering these advantages, reproducibility, and safety, we chose the lentivirus-mediated gene therapy system as an initial proof-of-concept in this study.

The spinal cord clip injury model used in this study is a clinically relevant model of chronic pain in SCI patients [57]. Rats in this model exhibit robust withdrawal responses to innocuous and noxious stimulation of their hind paws, consistent with the presence of allodynia and hyperalgesia. Below-level neuropathic pain is especially bothersome and difficult to treat in patients with both incomplete and complete SCI. This model has shown good predictive validity using a variety of pharmacologic agents, including those with some or no reported pain-reducing benefits in SCI pain patients [57].

Endomorphins inhibit nociceptive transmission in the spinal cord through μ-opioid receptors which are localized in neuronal circuits involved in processing of nociceptive information [27]. Despite a distinct anatomical distribution of endomorphins with EM-1 present mainly in the brain and EM-2 in the spinal region rich in μ-opioid receptors [58], both actively participate in nociceptive transmission after peripheral stimuli. Antinociceptive effects of the intrathecal injection of EM-1 or EM-2 were observed in models of peripheral nerve injury [18, 31].

NMDAR is another potentially promising target for enhancing analgesic activity. NMDAR in the superficial dorsal horn has been implicated as a major contributor to excitatory nociceptive transmission [59]. Substantial evidence has established that activation of the NMDAR in the spinal dorsal horn is essential for central sensitization [60, 61]. SHG was identified in a screen for compounds that enhanced adrenal transplant-mediated analgesia in rat pain models [62]. It is a stable synthetic analogue of histogranin with NMDA antagonist activity and has the potential to be delivered recombinantly on a long-term basis using emerging lentiviral technologies. Intrathecal administration of SHG blocks the hyperalgesia and allodynia produced in rats by intrathecal NMDA administration with no motor side effects [35, 63]. This finding is especially important from a clinical view, as the intrathecal administration of NMDA receptor antagonists, such as ketamine, has a promising role in patients that are poorly responsive to traditional analgesics [64]. The use of an NMDA receptor antagonist in pain therapy is often accompanied by motor deficits which limit their potential therapeutic value for neuropathic pain management. In the current study, deleterious motor side effects induced by the lentiviral injection encoding SHG were not observed. Similarly, in previous studies using bolus intrathecal SHG injection, locomotor side effects were not observed in contrast to other NMDA antagonists [17]. Using the clip compression model, rats develop a partial paralysis due to SCI with modest progressive recovery, as observed with the BBB test. The BBB test scores of animals injected with EMs and SHG lentiviruses were comparable to those without injections and with the animals injected with the control GFP lentivirus.

Behavioral tests for tactile, thermal and mechanical hypersensitivity revealed beneficial effects of combined injection of EMs and SHG on SCI induced pain. The analgesic effect was observed as soon as 1 week post injection for some modalities, and sustained for at least up to 7 weeks, when the behavioral study was terminated. Interestingly, only modest effects, at best, were observed with individual peptide vectors. This may be due to the local levels of available peptides that can be achieved by the viral vectors, perhaps insufficient for reducing neuropathic SCI pain. Previous findings in our lab have indicated that the blockade of spinal NMDA receptors alone, via bolus intrathecal injection of either SHG or another NMDA antagonist ketamine, is insufficient in reducing neuropathic pain symptoms in the SCI clip compression model [57]. Thus, it is perhaps not surprising that vector delivery of SHG alone was ineffective in reducing SCI pain in the current study. Both SHG and the EMs have reported effectiveness in numerous other pain models, although recent findings in our lab has shown that these effects are variable depending on the selected models and outcome measures [18]. It is likely that the labile nature of small neuropeptides limits their time course and efficacy; thus a gene therapy approach may be ideal in overcoming this limitation.

Although the intrathecal injection of endomorphins alone have not been evaluated in the SCI clip compression model, a possible explanation for their limited effectiveness alone using the viral vector approach in the current study is the potential development of tolerance to continued exposure to these mu-opioids mediated by sustained delivery from transduced spinal cells. If this is the case, results of this study provide strong support for both the additive effects and reduced tolerance development of this combination strategy. In contrast to the singly delivered peptides, when co-delivered using the combined SHG and EM viral vectors, potent analgesic effects, once initiated, were maintained without decrement through the study duration. The contributions of both the EMs and SHG in mediating the sustained analgesic effects of the viral vectors is supported by the reversal using intrathecal injection of opioid (naloxone) or NMDA antagonist (anti-SHG).

The time of delivery of therapeutic compounds in SCI model is an important issue because delayed onset of the pain treatment after SCI is clinically more relevant than early interventions. In this study we showed that the delayed treatment using viral vector-mediated delivery of analgesic peptides could still be effective in alleviating SCI neuropathic pain. The reduced pain responses in animals receiving intraspinal injection of EMs and SHG 6 weeks after SCI were similar to the reduced pain responses of animals receiving the injections on the second week after SCI. This suggests that delayed injection of lentiviral vectors encoding the appropriate analgesic peptides can still alleviate chronic pain in SCI models.

## Conclusions

In conclusion, the present series of studies provides evidence that SCI-induced neuropathic pain may be controlled by intraspinal gene therapy that drives the production and release of an NMDA antagonist peptide and endomorphins.

Given that this type of neuropathic pain is especially difficult to treat, the success of this gene therapy approach is significant [65]. The long term effect of intraspinal administration of lentiviral vectors encoding SHG and endomorphins provide support for the concept that such combined therapy can indeed control enhanced pain states without overt adverse effects. The prolonged analgesic effects observed here also suggest the lack of tolerance development using this strategy, an important feature for potential clinical application in long term pain management.

## Methods

### Construction of the lentiviral vectors encoding SHG and EMs

The generation and characterization of the SHG construct has been previously published [44]. Similar approaches were used for constructs encoding endomorphins [66]. Briefly, peptide sequences of EM-1 and EM-2 were reverse translated. Sense and anti-sense oligonucleotide primers (Sigma Genosys, Miami) were synthesized and annealed to produce double stranded DNA (dsDNA) sequence coding flanked by Bgl II/Sal I half sites, encoding either EM-1 or EM-2 As endomorphins are endogenously amidated, these oligos include a glycine before the stop codon. Peptides with extended glycine are substrates for the amidation complex.

These constructs were subcloned into lentiviral vectors and high titer purified recombinant lentiviruses encoding either SHG, or EM-1 or EM-2 were generated by the in house viral vector core. Briefly, viral stocks were produced by co-transfecting SHG/pRRL or EM/pRRL with packaging vectors in 293 T cells. Three packaging plasmids (self-inactivating vector plasmid, third generation packaging plasmid and an envelope plasmid, pLP1, pLP2, and pLP/VSVG) supply the structural and replication proteins *in trans* that are required to produce the lentivirus. Control lentiviruses were generated at the same time by co-transfecting the 293 T cells with the identical 3 packaging plasmids and pRRLs in PPT.CMV.WPRE containing the 720 bp GFP (GFP/pRRL). Vector batches were tested for the absence of replication-competent virus by monitoring p24 antigen expression in the culture medium of transduced 293 T cells for 2 weeks as an additional safety check. Viral p24 antigen concentration was determined by ELISA (PerkinElmer, Boston, MA). In all cases tested, p24 was undetectable and transducing activity was expressed in transducing units (TU). Nerve growth factor signal sequence gene (preproNGF-β) was used for the recombinant peptide to be targeted to the secretory apparatus of the cells and consequently get secreted as previously reported [44, 66, 67]. All construct sequences were confirmed by the Genewiz sequencing facility using gene-specific primers (sense primer 5’-TTGGATCC GCGTAATGTCCAATGTTGT-3’ and anti-sense primer 5’-AGTCTAGACA TAAAGCCTCCGTATACT-3’) to verify presence and expected sequence of each recombinant gene and in polymerase chain reaction as previously published [66].

In order to visualize transfected cells in vitro, some of the SHG and EM-1 and EM-2 constructs were subcloned into the lentiviral vector in the frame with a reporter gene mRFP using the protocol described above.

The following types of viral vectors were used:

1. The control viral vector containing only the GFP (green fluorescent protein) gene (Lenti/GFP).
2. The viral vector containing SHG gene that encodes the serine histogranin (Lenti/SHG with mRFP for immunohistochemical detection.
3. The viral vector containing endomorphin gene that encodes EM-1 (Lenti/EM-1 with mRFP for immunohistochemical detection).
4. The viral vector containing endomorphin gene that encodes EM-2 (Lenti/EM-2 with mRFP for immunohistochemical detection).

### Transgene viral expression in vitro

The schematic in Figure 1 shows the features of the lentiviral construct as previously published [66]. SH-SY5Y cell lines were grown in DMEM/F12 medium (Invitrogen, Carlsbad, CA) supplemented with 10% fetal bovine serum (FBS) and antibiotics. At 70% confluency the cells were transduced with Lenti/endomorphins or lenti/SHG viruses (10^8^ transducing units per ml (TU/ml) based on viral coat protein p24 ELISA assay. Five days after transduction the cells were fixed for 10 minutes with 4% paraformaldehyde, permeablized with phosphate buffered saline (PBS) with 0.1% Tween-20 (PBST) for 10 minutes and then stained for endomorphins or SHG by standard immunofluorescence protocols using antibodies against EM-1/EM-2 (1:300, Genetex) and SHG (1:1000 , 21st Century Biochemical). Briefly, cells were incubated in 5% normal goat serum and then in primary antibody for one hour in each step. After washing with PBS, secondary antibodies (1:300 dilution, Alexa-fluor 594 or 488 goat anti-rabbit, Invitrogen) were added for 30 minutes. Cells were washed in PBS and incubated with 120 ToPro/DAPI for 10 min. Finally, the slides were coverslipped with mounting medium. Primary antibody was withheld from control slides to determine background due to non-specific staining.

### Animals

Male Sprague–Dawley rats weighing 150–170 g at the initiation of the studies were used. All animal procedures followed NIH guidelines and were approved by the University of Miami Institutional Animal Care and Use Committee.

### Surgeries

All the surgical procedures were performed under aseptic technique in a special room design for surgery purposes. For spinal cord injury, after anaesthesia with mixture of isofluorane and O_2_, a laminectomy was performed at T6–T8. The spinal cord was compressed by a micro-vascular clip (Harvard Apparatus, MA) for 1 min. After the one minute compression, the clip was removed carefully (care was applied not to damage the dura or spinal nerves), the muscles sutured with 4–0 chromic gut (Ethicon Inc., Somerville, NJ) and wound clips were used to close the skin. From the day after surgery the bladder was manually expressed in the morning and afternoon until the animals were able to express their bladders independently.

Based on the analgesic effect of peptide SHG and morphine [17] and the effectiveness of a ratio of synergistic analgesic peptide combination [68] we chose to test the mixture of EM-1/EM-2 and SHG at the same ratio i.e., 3:3:1. Animals with 2-week old SCI were randomly allocated to different viral vector-injected groups. The control group received lentivirus encoding GFP. In a separate group of animals, to explore whether delayed vector treatment could still be effective on the alleviation of pain, the mixture of endomorphins and SHG was injected six weeks after SCI. For all injections, spinal cords at the level of L3/L4 were exposed by laminectomy and each animal received four injections of 20 ng/μl of lentivirus (10^8^ TU/ml based on p24 ELISA ) bilaterally (two per side) in the spinal cord 1 mm apart. For animals receiving combined vector injections, since the mixture of EM-1/EM-2 and SHG were done at ratio 3:3:1, the individual doses in the mixture were 8.6 ng EM1, 8.6 ng EM2, and 2.8 ng/ μl. Injections were done stereotaxically (Kopf) in a volume of 1 μl/site (0.5 μl/min) at a depth of 0.3 mm from the dorsal border and 0.7 mm from the midline using a glass pipette attached to a 10-uL Hamilton syringe mounted on a microinjector. To prevent backflow, the needle was kept in its place for one minute after the termination of the injection. To maximize reproducibility, injections were performed by only 1 surgeon. The overlying muscles were sutured by 4–0 chromic gut and the skin was closed with wound clips. Animals were allowed to recover at 37°C for 24 h, after which time they were returned to the animal care facility.

### Behavioral tests

All the behavioral tests were done by evaluators who were unaware of experimental groups. Animals were tested before spinal cord injury (SCI), one week after SCI, before intraspinal injection of the vectors and then every week after for seven weeks.

### Motor Function Recovery test

The Basso-Beattie-Bresnahan (BBB) test was used for evaluation of motor behavior [42]. Rats were placed in the center of an open-field area with a 4 foot diameter, and the behavior of the animals was observed for a 4 min test period by two individuals blinded to the treatment. The scale was designed to reflect motor rating scores. Briefly, the BBB scale involves closely monitoring limb movement, weight-bearing capability, coordination, and gait. The scores range from 0 to 21. Zero indicates complete paralysis without joint movement and 21 indicates normal locomotion with full coordination and proper gait, movement at all joints, full weight support, and appropriate limb, body and tail positioning.

### Sensory tests

For the assessment of mechanical and cold allodynia the rats were placed on a metal mesh covered with a plastic dome (13 × 17 × 28 cm) for at least 20 minutes before testing. The thresholds for mechanical allodynia were measured with a series of von Frey filaments. The filaments were applied to the plantar skin of the hindpaw and bent slightly. Withdrawal of the paw, usually followed by licking of the paw or body movement, was recorded as a positive response. Eight specific calibrated von Frey filaments were used via the up-down method to determine the withdrawal threshold. The filaments with 0.25 g and 15 g force were selected as lower and upper limit respectively. A hind paw withdrawal from the filament led to the use of the lower force filament. A lack of response led to the use of the next higher force filament. If the strongest hair did not elicit a response, the threshold was recorded as 15 g. Both hind paws were tested and about 5 min separated testing of the opposite paw.

**Cold allodynia** was measured as the number of foot withdrawal responses after application of an acetone drop to the plantar surface of the paw. Usually the normal rat did not respond to acetone application, but the injured rats showed nociceptive responses, such as foot shaking or biting. The testing was repeated five times with an interval of approximately 3–5 min between each test. The response frequency to acetone was expressed as a percent response frequency ([number of paw withdrawals/number of trials] × 100).

**Thermal nociceptive threshold** was assessed with the method first described by Hargreaves et al. [69] using the Plantar analgesiometer (Ugo Basile model 7371). At the beginning of each testing day, animals were placed in test cages for acclimation 30 min before. The testing device used light from a slide-projector lamp passed through a filter that allowed passage of only infra-red radiation. At the beginning of each test the heat source was positioned under the middle of the right or left hind paw. Once in position, the heat source was activated, which slowly heated the paw. On paw withdrawal, a photo-cell automatically shut off the heat source and recorded the time to withdrawal.

To avoid causing a thermal injury in the event that the animal did not withdraw its paw voluntarily, there was an upper limit cut off of 25 sec after which the heat was automatically terminated. If paw movement subsequent to stimulus initiation appeared to be related to grooming or locomotion, that trial was repeated after a lapse of 1 min. Testing was repeated 3 times for each paw with at least 5 minutes interval between the trials and the average of three trials per paw was used to report the final withdrawal latency.

**Mechanical hyperalgesia** was measured using increased mechanical pressure applied to the rat paw with an analgesiometer (Ugo Basile) [43]. The rats were wrapped in a towel and an increasing force (48 g/s) was applied to the plantar surface of the hind paw until the rat reacted with vocalization or started to exhibit the flight or struggle response. The apparatus terminated at 1000 g (25 in scale units) in the absence of a response. On all testing days the measurement of thermal sensitivity was followed by the measurement of sensitivity to mechanical stimuli.

To assess pharmacologic mechanisms, some animals received intrathecal injection of anti-serine-histogranin (10 μl, 0.5 μg/ml) or naloxone (1 μg in 10 μl), and were tested for mechanical and cold allodynia before and after injection.

Direct transcutaneous intrathecal injections were used by inserting a 25 G needle connected to a 25 μl Hamilton syringe into the tissues between the dorsal aspects of L5 and L6 perpendicular to the vertebral column. This site was selected in order to reduce the possibility of spinal damage and increase the intervertebral accessibility. In this location the spinal cord terminates and the cauda equina begins. Sudden lateral movement of the tail was used as an indicator of successful puncture. Naloxone was dissolved in saline and injected in a volume of 10 μl. After injection the syringe was held in position for a few seconds and then removed slowly to avoid any outflow of the drug. Cold and mechanical allodynia assessment was done 15 min after injection.

### Immunohistochemistry

Rats were deeply anesthetized with a mixture of Ketamine and Xylazine (100 and 40 mg/kg respectively, ip) and perfused transcardially with saline, followed by 4% paraformaldehyde in 0.1 mol/L phosphate buffer at pH 7.4. Spinal cords were removed and immersed in the same fixative overnight in 4°C, and transferred to 25% sucrose in phosphate buffer for cryoprotection. Twenty-micrometer-thick sections were rinsed in phosphate-buffered saline (PBS) and Tris buffer saline, incubated in filtered 2% sodium paraperiodate in TBS for 20 min. Sections were incubated in 5% normal goat serum (NGS) for 30 min, then overnight with primary antibody anti-SHG (1:1000, 21st century biochemical), anti-NeuN (1:200, Chemicon), anti-GFAP (1:200, Sigma). There is no commercial source for anti-histogranin or anti-serine histogranin antibodies; therefore, a custom antibody for SHG was generated for us with 21st Century Biochemicals. A synthetic SHG peptide was used to immunize rabbits. Based on our published data [44, 66], anti-SHG antibody showed a dose dependent reaction with the synthetic SHG peptide. The specificity of the reaction was supported by the lack of reaction of anti-SHG antibody (high titers) with an irrelevant peptide, EM-1 [44] or anti-EM-1 and 2 antibody (3 μg/ml, Genetex) in PBS + % 0.4Triton X (PBST) at 4°C. After washing with PBS, sections were incubated in the appropriate secondary antibody (1:300 dilution, Alexa Fluor 594 and 488 goat anti-rabbit and Alexa Fluor 488 goat anti-mouse, Invitrogen) for 2 h. Tissue sections were washed in PBST and 120 ToPro/DAPI was added for 10 min. Finally, the sections were coverslipped with fluorescence mounting medium.

### Immunodot-blot

A sample of cerebrospinal fluid (CSF) of animals injected with the vectors was collected from the cisterna magna. Briefly, the atlanto-occipital membrane between the occipital bone and the upper cervical vertebra was exposed by a small incision in the skin and overlying muscle. A needle connected to an insulin syringe was inserted slightly into the membrane and a clear CSF sample was drawn without any blood contamination. The immunodot-Blot procedure was adapted from Abcam Company. The stock solution of EM-1, EM-2 and SHG peptide were prepared with saline. Serial dilution of each peptide was prepared in concentrations ranging from 0.25 μg/μl to 0.008 μg/μl for SHG and from 1 μg/μl to 0.03 μg/μl for endomorphins. 2 μl of each sample was spotted on the 0.22 μ pore-size nitrocellulose membrane separately. Serial dilution of the CSF of animals injected with Lenti/SHG and EM-1/EM-2 was prepared and 2 μl of each samples spotted on the membrane. After air drying at room temperature, the membranes were incubated in blocking serum containing 5% bovine serum albumin (BSA) in Tris-buffered saline with 0.4% triton (TBST) for one hour before being transferred into the primary antibody. The same primary antibodies with the same dilution in the immunohistochemistry protocol were used. Membranes were incubated in primary antibody for 30 min before washing with PBST for three times 5 min each. Membranes were incubated in secondary antibody conjugated with horse-radish peroxidase (HRP) (1:10000 anti-rabbit IgG-HRP Santa Cruz Biotechnology) for 30 minutes at room temperature. After washing with PBST, enhanced chemiluminescent substrate (ECL, Thermo Scientific) was used for detection of HRP. Membranes were incubated in ECL for 1 min and then exposed to X-ray film in the dark room.

### Statistical analysis

The results are presented as mean ± SEM. Statistical analysis of data was carried out using GraphPad Prism. Data were compared among groups with two-way analysis of variance with repeated measures ( RM ANOVA) followed by the Bonferroni post hoc test and by *t-test*. Statistical significance was assumed when *P* < 0.05.

## Abbreviations

AAV: Adeno associated virus
BBB: Basso, Beattie, Bresnahan, and Basso locomotor rating scale
BSA: Bovine serum albumin
CCI: Chronic constriction injury
cDNA: Complementary DNA
CSF: Cerebrospinal fluid
DRG: Dorsal root ganglia
EM: Endomorphin
GFP: Green fluorescent protein
HRP: Horseradish peroxidase
HSV: Herpes simplex virus
NMDA: N-methyl-D-aspartate
NMDAR: NMDA receptor
PBS: Phosphate buffered saline
PBST: Phosphate buffered saline with 0.4% Triton X
SCI: Spinal cord injury
SHG: Serine^1^-histogranin
TBST: Tris buffered saline with 0.4% Triton X.
